# In Your Face: Risk of Punishment Enhances Cognitive Control and Error-Related Activity in the Corrugator Supercilii Muscle

**DOI:** 10.1371/journal.pone.0065692

**Published:** 2013-06-26

**Authors:** Björn R. Lindström, Isak Berglund Mattsson-Mårn, Armita Golkar, Andreas Olsson

**Affiliations:** 1 Department of Clinical Neurosciences, Karolinska Institutet, Stockholm, Sweden; 2 Stockholm Brain Institute, Stockholm, Sweden; University of Gent, Belgium

## Abstract

Cognitive control is needed when mistakes have consequences, especially when such consequences are potentially harmful. However, little is known about how the aversive consequences of deficient control affect behavior. To address this issue, participants performed a two-choice response time task where error commissions were expected to be punished by electric shocks during certain blocks. By manipulating (1) the perceived punishment risk (no, low, high) associated with error commissions, and (2) response conflict (low, high), we showed that motivation to avoid punishment enhanced performance during high response conflict. As a novel index of the processes enabling successful cognitive control under threat, we explored electromyographic activity in the corrugator supercilii (cEMG) muscle of the upper face. The corrugator supercilii is partially controlled by the anterior midcingulate cortex (aMCC) which is sensitive to negative affect, pain and cognitive control. As hypothesized, the cEMG exhibited several key similarities with the core temporal and functional characteristics of the Error-Related Negativity (ERN) ERP component, the hallmark index of cognitive control elicited by performance errors, and which has been linked to the aMCC. The cEMG was amplified within 100 ms of error commissions (the same time-window as the ERN), particularly during the high punishment risk condition where errors would be most aversive. Furthermore, similar to the ERN, the magnitude of error cEMG predicted post-error response time slowing. Our results suggest that cEMG activity can serve as an index of avoidance motivated control, which is instrumental to adaptive cognitive control when consequences are potentially harmful.

## Introduction

Cognitive control is engaged to match behavior to goals when well-established responses are contextually inappropriate, or when several possible responses conflict [1], [2]. For example, if you are used to cars driving on the right side of the road, and find yourself visiting England where traffic is left-handed, you need to cognitively control your habitual impulse to look to the right when crossing the street [2]. In situations like this, the failure to control your behavior can be fatal. However, the need for cognitive control is not only dependent on your goals. It is likely to be directly related to the perceived risk of harm if one fails to control behavior; on a sparsely travelled country road, control might be less needed than in the heart of London, where the risk of harm following control failure is much higher.

Research on cognitive control has commonly emphasized the role of task goals in guiding adaptive behavior, and less is known about how the *consequences* of flawed control affect behavior. This is surprising, because considering the consequences of one's behavior can be crucial to survival in many situations [3]. In fact, the consequences of control-demanding real-world behaviors are seldom neutral (e.g., looking in the wrong direction while crossing the street in England or acting inappropriately in a social situation), and the need for controlled behavior is likely to increase as a function of the risk of aversive consequences following control failure. A better description of the relationship between cognitive control and the risk of aversive consequences of control failure is of importance for our understanding of both normal social and emotional functioning, and of stress- and anxiety-related psychiatric disorders [4] , as well as disorders characterized by control failure (e.g., relapse in addiction) [5].

The aim of the present research was twofold: First, we aimed to describe how the perceived risk of punishment following control failure impact cognitive control (henceforth *avoidance motivation*). To address this question, we examined performance in a response conflict task, where mistakes incurred an increased risk of being punished by aversive electric shocks. Secondly, to better describe the underlying processes, we investigated electromyographic activity in the corrugator supercilii muscle (cEMG) as a novel index of the integration of cognitive control and avoidance motivation.

### Motivation Affects Cognitive Control

Recently there has been a surge of interest in characterizing the interaction between motivation and cognitive control [6]–[11]. Behaviorally, motivation has been shown to reduce response conflict [12] and improve performance [7]. The primary focus of these studies has, however, been in the domain of reward motivation. Reward-oriented [13] and aversion-driven learning [14], [15] engage partially separable neural substrates and involve different neurotransmitter systems [16], [17], suggesting that the impact of reward-oriented and avoidance-based motivation on cognitive control likewise might differ. Recent work examining the impact of motivation to avoid losing money on cognitive control showed that loss of money modulated performance in the Go/No-Go task, by inducing slower responses and fewer commission errors relative to a control condition [18]. These results suggest that punishment with a secondary reinforce can result in a more cautions response strategy. In contrast, primary aversive stimuli, such as electric shocks, have been shown to have detrimental effects on cognitive control [19], [20]. Importantly, because these studies have typically been aimed at modeling the effects of general anxiety on behavior, the delivery of shocks has been unrelated to performance. Taken together, earlier studies do not speak directly to the effect of the motivation to avoid primary punishment through controlled behavior, because they have used either secondary (e.g., money) reinforcers or primary (e.g., electric shocks) reinforcers unrelated to performance. Our aim was to examine the impact of avoidance motivation on controlled behavior in situations where flexible control over behavior is needed to avoid potentially dangerous physical consequences. Following this, our task included the use of a primary reinforcer; an electric shocks as a consequence of control failure.

### Psychophysiological and Neural Correlates of Motivated Cognitive Control

Recently, Shackman and colleagues described a brain-based framework for how cognitive control and negative emotion interacts; the Adaptive Control Hypothesis (TACH) [21]. Based on an extensive meta-analysis of fMRI studies that demonstrated overlapping activation to cognitive control (see [22] for a review), negative emotion [23] and pain [24] in the anterior mid cingulate cortex (aMCC), TACH postulates that this regions integrates information about negative reinforcers (e.g., pain) arriving from cortical and subcortical afferents (e.g., insula, striatum, amygdala), to bias behavioral selection away from punishment. The bias of behavioral selection is foremost needed in demanding or potentially dangerous situations, for example, when the consequences of action are uncertain (e.g., probabilistic learning), multiple conflicting response alternatives are active, or when failure of an intended action is associated with potential punishment [21]. By identifying overlap between cognitive control, negative emotion and pain at the level of functional anatomy and linking this overlap to functional integration of cognitive and affective processes, TACH led us to predict that motivation to avoid aversive consequences would have important consequences for cognitive control. Based on research on the neural overlap between cognitive control and negative affect in the aMCC, we also predicted that activity in the frowning muscle, corrugator supercilii, would be a novel index of the integration between cognitive control and avoidance motivation. The aMCC contributes to facial expressions of negative affect in primates, by projecting to the muscles of the upper face (e.g., the corrugator supercilii and frontalis majoris) via the brainstem facial nucleus [25], [26]. The corrugator supercilii is one of the main muscles involved in negative facial expressions, such as anger or fear, in both humans and non-human primates [27], [28]. Electryomyographic activity in the corrugator supercilii (cEMG) activity is also elicited when subjects view aversive stimuli [29], negative facial expressions [30] and experience physical pain [31]. Interestingly, evidence also suggests that cEMG activity is sensitive to cognitive control demands, e.g., response conflict. For example, Schacht and colleagues reported prolonged cEMG activity on trials requiring inhibitory control in a Go/No-go task [32]. In concert, these studies suggest that the cEMG might serve as a novel index of the integration of cognitive control and avoidance motivation.

Additional support for this proposal comes from research on the properties of a well-established index of cognitive control: the Error-related Negativity (ERN) ERP component elicited by error commissions in experimental tasks [33]–[35]. Importantly, the ERN is amplified as a function of the severity of the consequences of errors, such as monetary punishment and social evaluation [36]. Recently, Riesel and colleagues [37] showed that punishing errors in a flanker task with an aversive noise also amplified ERN amplitude. Based on such findings, researchers have proposed that the ERN might index “affective” qualities of error monitoring [38] in addition to the traditional emphasis on cognitive control [33]. This integration between cognitive control and affect is well-explained by TACH, and further supported by localizing the neural generator of the ERN to the ACC/aMCC [38]. The ERN thereby exemplifies the tight coupling between a *process* (cognitive control to avoid costly errors) and its *neural underpinnings* (the aMCC) described by TACH. Although we do not directly measure the ERN in the present study, we base our research question on a similar logic by looking for similarities in the response properties of the cEMG to those reported for the ERN. The impetus was to enhance our understanding of the *processes* underlying the integration of cognitive control and avoidance motivation by studying the cEMG signal.

### The Present Study

The aim of the present study was two-fold: (1) to characterize how avoidance motivation impacts cognitive control through a parametric manipulation of perceived *punishment risk*, and (2) investigate the cEMG as a novel index of this process by means of testing a set of specific hypotheses derived from recent work on the neural underpinnings of motivated cognitive control [21].

**Figure 1:**
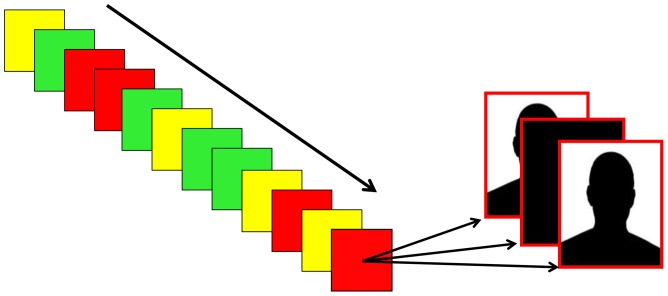
Figure 1. Illustration of the experimental task, which had a mixed 3 (Punishment Risk: No/Low/High) x Cognitive Conflict (Low/High) block/event design. The sequence of blocks (left) was randomized for each subject, with four blocks of each Punishment Risk level. Green color indicates No Risk blocks, yellow Low Rick blocks, and red High Risk blocks. A sequence of two example trials is shown to the right. Each block included 80 trials, where 75% was Low Conflict and 25% High Conflict. Note that images of actors from the Karolinska Directed Emotional Faces stimuli set were used in the actual experiment. For copyright reasons, these are represented by a silhouette.

We used a two-alternative forced choice version of the Go/No-Go task to manipulate response conflict (low/high) on a trial-to-trial basis (see Fig. 1). The task sets an infrequent (25%) response in conflict with a more habitual one (75%) [39]. To delimitate the effect of avoidance motivation from behavior directly induced by aversive reinforcers [17], [40], the perceived risk of punishment (henceforth *punishment risk*) was manipulated while we controlled for the actual amount of punishment delivered to the participants. Thus, unbeknownst to the participants, they always received a fixed number of electric shocks regardless of their actual performance level. Punishment risk was induced through a threat-of-shock procedure, in which participants were informed that response errors could be punished with mild electric shocks. Three levels of punishment risk were included (no risk, low risk, high risk), being the minimal parametric manipulation needed to capture non-linear effects of punishment risk. Importantly, no actual contingency between the number of errors and the number of electric shocks existed, which allowed us to draw conclusions about the effect of punishment risk in the absence of variability in the amount of punishment across the group.

#### Predictions: Behavior

As previously noted, the previous studies using threat-of-shock in relation to cognitive control have used procedures in which performance and shocks are unrelated with the impetus to model the effects of anxiety on performance. However, based on the studies where monetary rewards and punishments are contingent on performance [7], [10], [18], we predicted that performance should be facilitated during the punishment risk conditions relative to a neutral baseline. Furthermore, as error commissions are more likely during high cognitive control demands [41], the effect of punishment risk on performance should be most pronounced during the high response conflict condition.

#### Predictions: Corrugator Supercilii

Our strategy was to look for functional and temporal analogues between the cEMG and previously reported response properties of the ERN in the context of cognitive control and motivation (see [38] for a recent review). Based on the ERN and TACH [21], we systematically tested a set of specific predictions for the cEMG with reference to the theoretical properties of a signal that integrates cognitive control and avoidance motivation. Seven predictions were made (< > indicate ordinal relations in predicted cEMG amplitude): (1) high response conflict > low response conflict, (2) punishment risk > no punishment risk, (3) high punishment risk x high response conflict > all other combinations of punishment risk and response conflict, (4) error responses > correct responses, and (5) error responses during punishment risk > error responses during no risk. We also predicted that (6) cEMG following error responses would be functionally related to the slowing of response time (RT) on the following trial in a similar manner as previously reported for the ERN (post-error slowing [33]). Prediction 4–6 is based on the characteristics of the ERN, due to its role as a well-established index of motivated cognitive control and its origin in the ACC/aMCC.

To delimitate punishment risk from general shock-related anxiety in its impact on cEMG activity, we included a control group which performed an identical experimental task, with one critical exception. In contrast to the Experimental group, the Control group was explicitly informed that there was no contingency between task performance and the number of electric shocks they received. We predicted (7) that the Control group would not show potentiated cEMG following response errors, because error commission had no expected aversive consequences. We also measured the skin conductance level (SCL) across the task to be able to relate the impact of punishment risk on cEMG relative to a well-established physiological index of arousal.

In summary, we predicted that (i) behavioral performance should be enhanced by punishment risk, and (ii) cEMG activity should be sensitive to the combination of response conflict, punishment risk, and error commissions.

## Method

### Participants

Forty participants (19 female) with a mean age of 25.9 years (SD = 7.34), with normal or corrected-to-normal vision, were recruited through posters at the Karolinska Institutet campus and a local website advertising participation opportunities in scientific research. Participants received two movie-vouchers. All participants provided written consent. All procedures were approved by the ethics committee at Karolinska Institutet. Participants were randomly assigned to the Punishment Risk group (n = 22, 11 male), and the Control group (n = 18, 10 male). Four participants were excluded from the Control group, as they faultily reported a relationship between their performance and the number of received electric shocks (see below for details).

### Materials

The experiment was conducted on a desktop PC with a 19-inch cathode ray tube (CRT) monitor (with screen resolution 1280×1024 and refresh rate 85 Hz) placed in a sound-attenuated experimental chamber. Seventy grayscale faces (equally many men and women) with neutral facial expressions were selected from the Karolinska Directed Emotional Faces [42] set. The faces were surrounded by a colored frame (5 pixels wide). The aversive stimulus was a monopolar 100 ms DC-pulse electric stimulation (STM200; Biopac Systems Inc, www.biopac.com) applied to the participant's non-dominant forearm. The intensity of the electric shock stimulation was adjusted individually for each participant in a work-up procedure, based on the criterion “unpleasant but not painful” (mean voltage: 30.7, SD = 8.2)

### Physiological recordings

Electromyographic (EMG) activity of the left corrugator supercilii muscle was recorded using a BioPac (MP100; Biopac Systems Inc, www.biopac.com) device equipped with two miniature Ag/AgCl electrodes filled with electrolyte gel [43]. A third ground electrode was placed on the mid forehead, proximal to the hairline. The raw EMG signal (sample rate 1000 Hz) was amplified and filtered through a 28–500 Hz IIR band pass, followed by a 50 Hz IIR band stop. The signal was rectified and integrated with a time constant of 20 ms. Skin conductance level (SCL) were recorded with electrodes placed on the distal phalanges of the non-dominant hand (sample rate 250 Hz). The SCL was based on average activity across all blocks for each punishment risk level (shocks excluded).

### Behavioral Task

The participants performed a two-choice speeded gender decision task, and responded behaviorally using the left or right arrow keys on a standard keyboard (see Fig. 1 for overview). The probability of each target gender was asymmetric (75% male faces/25% female faces, or the reverse), giving a correspondingly asymmetric response ratio. This manipulation was based on the Go/No-Go task [44], where the high probability target induces a pre-potent tendency to respond, which has to be inhibited for low probability targets. However, the standard Go/No-Go task has apparent limitations due to the lack of a recorded response for the critical low probability (no-go) condition. For this reason, responses to both targets were collected. It should be noted that evidence from fMRI [45], [46] and computational modeling [47] indicate that response inhibition and response selection is highly related, or even overlapping, processes, suggesting that the task used in the present study and the standard Go/No-Go task are comparable. The 75% target condition is referred to as Low Conflict and the 25% target condition is referred to as High Conflict. The Low Conflict and High Conflict gender was counterbalanced across participants.

#### Punishment Risk

Punishment Risk was manipulated block-wise, with three levels; No Risk, Low Risk, and High Risk. For Low- and High Risk blocks, participants were instructed that any errors (both commissions and omissions) during the block might be punished with a mild electric shock *after* the block: *“Every time you make an error during a ‘LOW risk’ or a ‘HIGH risk’ round you may get a shock AFTER that round. The more error you make during a round, the greater risk for multiple shocks AFTER that round. ‘HIGH risk’ rounds give TWICE as many shocks as ‘LOW risk’ rounds.”* (Translated from Swedish).

Critically, the actual number of delivered shocks was identical for all participants. The intention was to manipulate punishment risk without introducing performance-contingent variability in the amount of experienced shocks across participants. Participants received 0–2 (uniform distribution) electric shocks after Low Risk blocks, and 2–4 (uniform distribution) electric shocks after High Risk blocks. Thus, no actual relationship between individual performance and number of shocks existed. Importantly, funneled interviews after the experiment showed that all 22 participants in the Punishment Risk group believed that there was a direct relation between their own performance and the number of shocks they received.

To fully assess the validity of the manipulation, 14 participants were randomly assigned to the Control group. The Control group performed the same experimental task and received the same number of electric shocks as the Punishment Risk group, but was explicitly informed that there was *no* contingency between performance and punishment. Four participants in the control group faultily reported a relationship between their performance and the number of shocks they received, and were therefore excluded from the analyses.

Both groups completed 12 blocks (4 blocks per Punishment Risk level) of 80 trials (total of 960 trials). Both block order (Punishment Risk) and trial order (Response Conflict) were fully randomized for each participant. A colored frame surrounding the target stimulus indicated Punishment Risk level (control group: ordinal amount of expected shocks) during the blocks (No Risk = green, Low Risk = yellow, High Risk = red). Target duration was 250 ms, followed by a 750 ms response period, and a 100–250 ms jittered inter-trial-interval.

### Data reduction and statistical analysis

#### EMG preprocessing and data reduction

The cEMG data was extracted in 100 ms time bins locked to the behavioral response on each trial using in-house software. Time-bins were extracted both prior (Pre) and following (Post) the behavioral response. The number of Pre-response relative to Post-response time-bins extracted on each trial was dependent on RT (e.g., for a trial with relatively long RT, more Pre-response time-bins and fewer Post-response time-bins were extracted, compared to a short RT trial). A 100 ms pre-stimulus baseline (mean cEMG amplitude) was subtracted from all time-bins to reduce slow signal drift and tonic level differences. Baseline measures below or exceeding 3 standard deviations were replaced and interpolated as an un-weighted average from the six adjacent baseline means. Mean and peak amplitude was computed for each time-bin.

EMG peak responses below or exceeding 3 standard deviations within each subject and time-bin were removed. The cEMG data was thereafter standardized (i.e., Z-transformed; scaled to mean 0 and standard deviation 1) across all-time bins within subject to enable comparison between time-bins. Within subject time-bins below or exceeding 5 standard deviations (the threshold was chosen to approximately reflect tail-end characteristics) were removed to reduce the impact of extreme outliers. All reported analyses were conducted on standardized peak amplitude data [43].

#### Statistical analyses

Generalized linear mixed models (GLMMs) were used for all analyses. The *lmer* function in the lme4 package for R was used for GLMM fitting [48]. Binary data (i.e., accuracy) was modeled with a logistic link function following a binomial distribution (GLMM) and continuous data (LMM) with an identity link function following a normal distribution. The goal in model construction was parsimony, where each model was aimed at addressing a specific hypothesis rather than to maximize explained variance. In contrast to the standard ANOVA analysis, the GLMM approach allow us to model accuracy on a trial-by-trial basis (rather than proportion correct) and flexibly incorporate both factorial and continuous predictors into the models and explicitly model both temporal correlations and random differences between participants, thereby increasing statistical power [49], [50].

All models included random intercept terms for each participant, and random slope adjustment by participant for each fixed effect predictor when supported by likelihood ratio tests, in order to find the maximum random effects structure supported by the data. In cases where model convergence failed, random slopes were included for the fixed effects with the largest effect sizes, in order of magnitude. Mean-centered trial number (1–960) and time-on-task (i.e., mean-centered RT) were included as covariates of no interest in all analyses to account for variance related to time on the macro (trial) or micro (RT) level. This random effect structure efficiently accounts for temporal dependencies in the data and overall random variability among participants [50].

Main- and interaction effects were evaluated with “Type II” analysis of deviance (i.e., analogous to Type II Sum of Squares ANOVA) tests based on the Wald statistic, in which the goodness-of-fit of nested models are compared against a χ^2^ distribution, using the *Anova* function in the car package [51] Note that estimation of main effects in the presence of higher-order interactions involving the main effect may be overestimated in Type II tests. Main effects in the presence of interactions are reported for completeness. The statistical significance of the simple main- and interaction effect parameters (i.e., if the parameter significantly differ from zero) was evaluated against the normal distribution, as no exact method for determining denominator degrees of freedom (df) currently exist for GLMMs [47]. The t-distribution and the normal distribution converge at high df [50], [52]. We also compared the *p*-values derived from the normal distribution with *p*-values based on likelihood-ratio tests for model comparison. These were identical or highly similar.

The asterisk operator is used as notation to indicate the factorial combination of terms (e.g., Conflict*Punishment Risk = Conflict+Punishment Risk+Conflict×Punishment Risk), and “x” to denote simple interaction terms. Data points below 200 ms and exceeding 1200 ms were removed for the analysis of RT, which only included correct trials. All graphs of cEMG results are displayed with T-transformed data (Z*10+50) to facilitate interpretation. Note that this scaling does not affect the results.

## Results

### Manipulation check: Skin Conductance Level

To asses if the punishment risk manipulation had an effect on overall arousal, SCL was analyzed with Punishment Risk (No/Low/High) as single predictor, which showed a strong main effect, χ^2^(2) = 61.16, *p*<.0001 (this effect was not related to individual differences in shock level). Simple effects showed a linear effect of Punishment Risk, where SCL was higher, relative to No Risk, for both Low Risk (*β* = 1.25, *SE* = 0.25, *z* = 4.97, *p*<.001) and High Risk (*β* = 1.96, *SE* = 0.25, *z* = 7.79, *p*<.001). The SCL was in addition higher for High Risk compared to Low Risk (*β* = 0.70, *SE* = 0.25, *z = *2.83, *p*<.01). These results indicate that punishment risk had a robust effect on arousal level.

### Behavior: Accuracy

**Figure 2:**
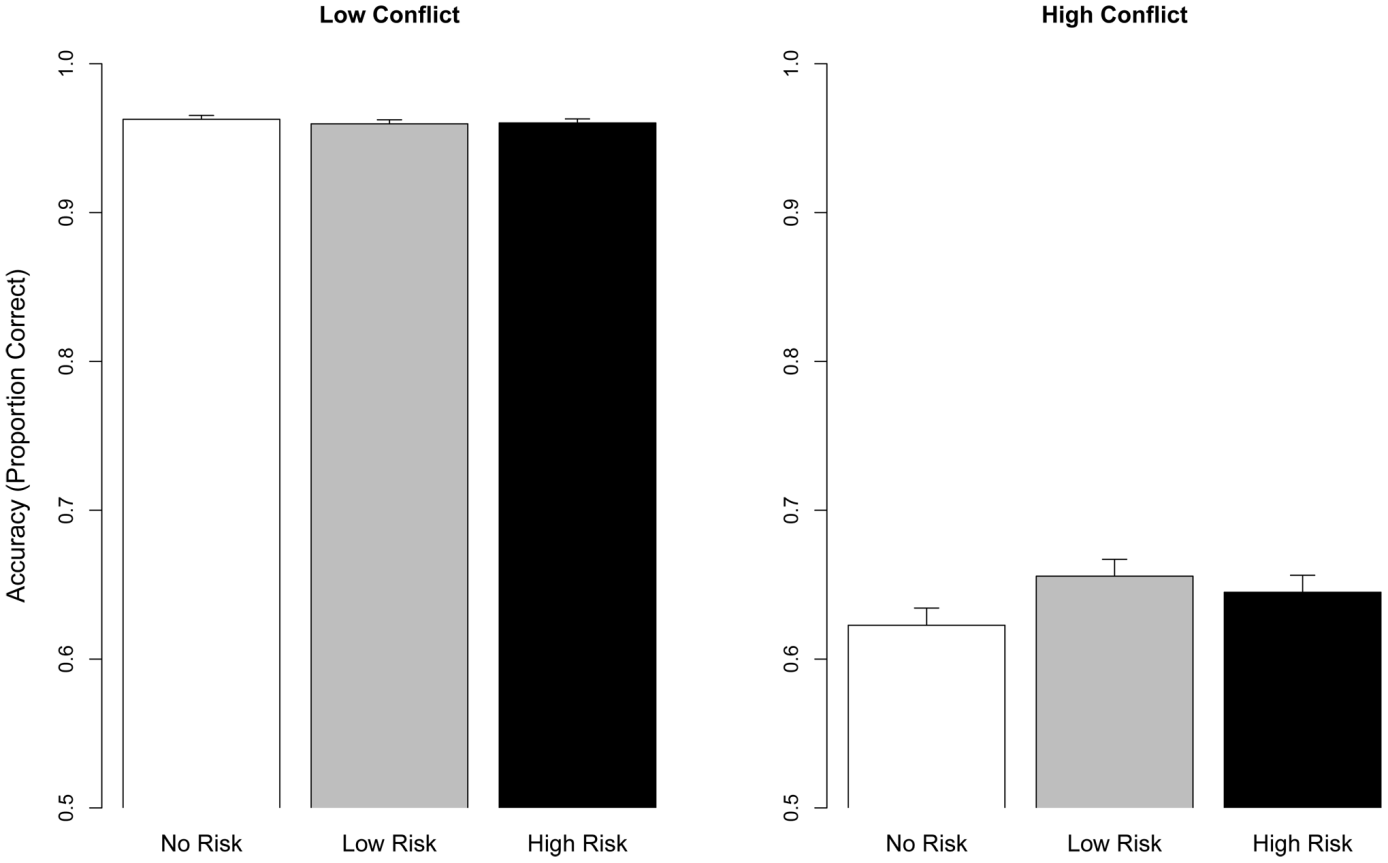
Performance accuracy as a function of Punishment Risk x Conflict. High and Low Conflict is plotted separately to visualize the Punishment Risk x Conflict interaction. Error bars denote SE.

Behavioral accuracy was analyzed in a GLMM with Punishment Risk (No/Low/High) * Conflict (Low/High) terms. The analysis showed a strong main effect of Conflict (χ^2^ (1) = 197.23, *p*<.001), no main effect of Punishment Risk (χ^2^ (2) = 1.30, *p* = .522), but a significant Conflict×Punishment Risk interaction (χ^2^ (2) = 6.96, *p* = .031) (see Fig. 2). Simple effects showed a cross-over interaction, where Low Risk decreased accuracy at Low Conflict (*β* = −0.29, *SE* = 0.12, *z* = −2.48, *p* = .013) compared to No Risk. However, this was reversed during High Conflict, where Low Risk attenuated the effect of High Conflict (see Fig. 2) (Low Risk×High Conflict: *β* = 0.39, *SE* = 0.15, *z* = 2.63, *p* = .008). No pair-wise contrasts were significant within either level of Conflict (*p*s>.11). Thus, Low Risk impaired performance when cognitive control demands were low, but enhanced performance when the cognitive control demands were high. High Risk did not reliably differ from either No Risk or Low Risk.

To formally characterize the shape of the relationship between Punishment Risk and accuracy, a polynomial model was inspected. A negative sign for the quadratic term indicate that accuracy is a concave function of Punishment Risk, i.e., the relationship has an inverted-U shape, while a positive quadratic term indicate that the relation is convex. The model showed that the effect of Punishment Risk was convex during Low Conflict (Low Conflict×Punishment Risk∧2: *β* = 0.19, *SE* = 0.08, *z* = 2.39, *p* = .017), and concave during High Conflict (High Conflict×Punishment Risk ∧2: *β* = −0.23, *SE* = 0. 1, *z* = −2.20, *p* = .028). In summary, Punishment Risk had an inverted-U like effect on accuracy during High Conflict, while the reverse was true during Low Conflict. This pattern of results was partially predicted, i.e., the enhancing effect of Punishment Risk on performance during High Conflict. However, this effect was most pronounced for Low Risk (note however that High Risk did not differ significantly from Low Risk).

### Behavior: Response Time

**Figure 3:**
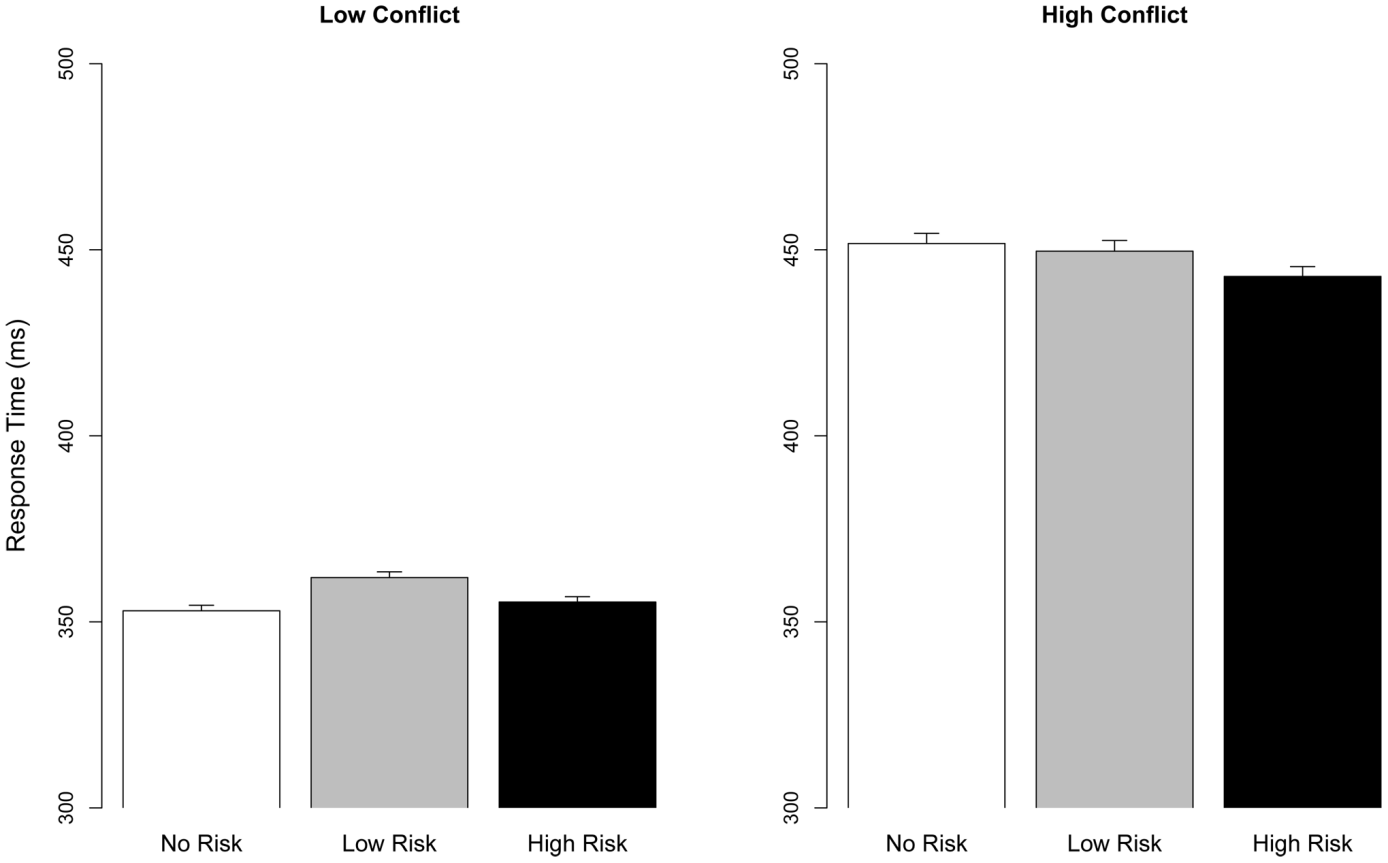
Response time as a function of Conflict and Punishment Expectancy. High and Low Conflict is plotted separately to visualize the Punishment Risk x Conflict interaction. Error bars denote SE.

Response time (RT) was analyzed with a LMM with Punishment Risk (No/Low/High) * Conflict (Low/High) terms. There was a main effect of Conflict (χ^2^ (1) = 197, *p*<.001), no main effect of Punishment Risk (χ^2^ (2) = 3.39, *p*>.1), which was qualified by a significant Punishment Risk×Conflict interaction (χ^2^ (2) = 8.49, *p* = .003) (See Fig. 3). This interaction reflected slightly slower RT for Low Risk during Low Conflict (*B* = 8.21, *SE* = 3.52, *z* = 2.33, *p* = .019) as compared to No Risk, whereas the detrimental effect of High Conflict was attenuated for Low Risk (Low Risk×High Conflict: *β* = −10.60, *SE* = 3.78, *z* = −2.82, *p* = .004), and for High Risk (High Risk×High Conflict: *β* = −7.88, *SE* = 3.79, *z* = −2.078, *p* = .019) relative to No Risk.

Similar to the effects on accuracy, Low Risk impaired performance by causing response slowing during Low Conflict compared to No Risk and enhancing it during High Conflict by speeding up RT relative to No Risk. High Risk specifically speeded up RT during High Conflict.

### Corrugator EMG: Prediction 1–3

To address Predictions 1 (high response conflict > low response conflict), 2 (Punishment Risk > no Punishment Risk), and 3 (high punishment risk x high response conflict > all other combinations of punishment risk and response conflict,) we fitted the corresponding three models; (i) Conflict (Low/High), (ii) Punishment Risk (No/Low/High), and (iii) Conflict (Low/High) * Punishment Risk (No/Low/High) to average EMG peak amplitude from all time-bins (899 ms Pre-response to 599 ms Post-response).

#### Prediction 1

There was no main effect of Conflict, (χ^2^ (1) = 1.62, *p* = .43 (*β* = 0.02, *SE* = 0.02) on average cEMG peak amplitude over the whole trial. Because the neural indices of response conflict are most apparent prior to the response on correct trials [39], we conducted a fine-grained analysis of cEMG limited to the Pre-response period for correct trials. This analysis supported the prediction, in showing the predicted effect of conflict on cEMG. The model, Conflict (Low/High) * Time-bin (899-800 ms Pre-response to 99-0 ms Pre-response [coded as 0∶8]), showed a Conflict x Time-bin interaction, χ^2^ (1) = 7.93, *p* = .005. This interaction was attributed to higher cEMG amplitude at the earliest Time-bins (i.e., at the intercept) for High relative to Low Conflict (*β = *0.10, *SE* = 0.05, *z* = 2.08, *p* = .037), and this difference declined linearly with time toward response onset (High Conflict x Time-bin: *β = *−0.02, *SE* = 0.007, *z* = − 2.82, *p* = .005). There was no effect of Time-bin on cEMG during Low Conflict (*z* = 0.13). These results suggest that cEMG during High Conflict reflects within-trial conflict resolution. Punishment risk showed no interaction with Conflict in the Pre-response period.

If pre-response cEMG actually reflects within-trial conflict resolution during High Conflict, one would expect cEMG amplitude to be predictive of behavioral accuracy. To directly test this hypothesis, we fitted a logistic GLMM to response accuracy. The model, (Conflict (Low/High) * cEMG * Time-bin (899-800 ms Pre-response to 99-0 ms Pre-response [coded as 0∶8]), showed a cEMG x Time-bin interaction (χ^2^ (1) = 7.04, *p* = .007) and a Conflict x cEMG x Time-bin interaction (χ^2^ (1) = 4.49, *p* = .033), which together showed that Pre-response cEMG amplitude early in the intra-trial time-course significantly predicted accuracy (*β = *0.56, *SE* = 0.16, *z* = 3.56, *p*<.001) during High Conflict and that the effect was completely abolished during Low Conflict (cEMG x Low Conflict interaction: *β* = −0.57, *SE* = 0.26, *z* = −2.23, *p* = .026). As above, the positive relation between cEMG and accuracy during High Conflict declined with temporal proximity to the response (cEMG x Time-bin interaction: *β* = −0.08, *SE* = 0.03, *z* = −2.23, *p* = .026). Thus, Pre-response cEMG activity was amplified by High Conflict, and this activity significantly predicted performance accuracy. These effects was most prominent at trials with longer RT, i.e., at the earliest time-bins.

#### Prediction 2

There was a statistical trend towards a main effect of Punishment Risk, χ^2^ (2) = 5.61, *p* = .061. Providing partial support for prediction 2, simple effects showed that while EMG amplitude during Low Risk was not clearly differentiated from No Risk (*β* = 0.09, *SE* = 0.07, *z* = 1.44), EMG amplitude was higher during High Risk than No Risk (*β* = 0.13, *SE* = 0.07, *z* = 2.09, *p* = .038). Thus, High, but not Low, Punishment Risk amplified cEMG.

#### Prediction 3

**Figure 4:**
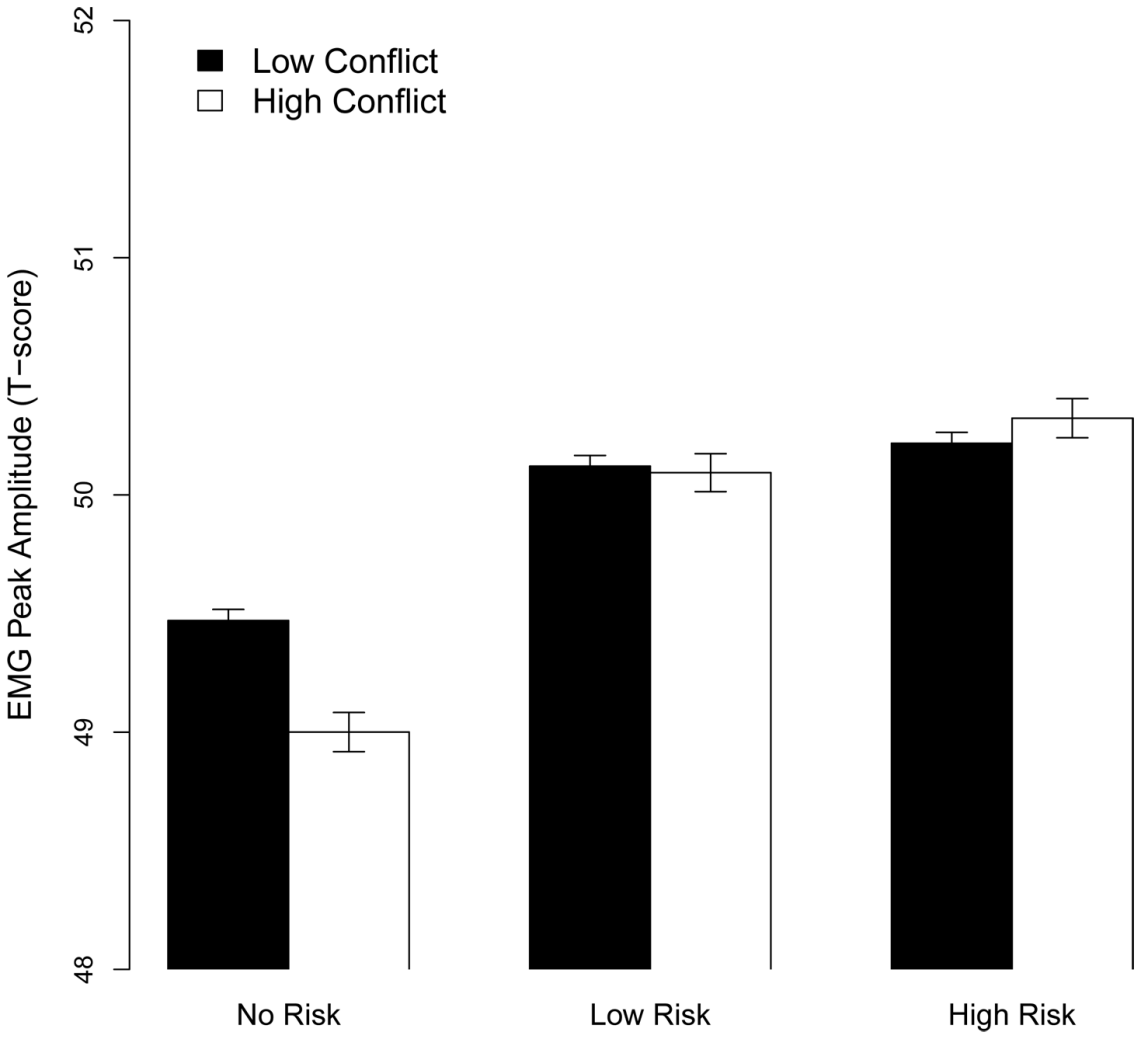
The effect of Punishment Risk x Conflict on cEMG (averaged over the whole trial). Error bars denote SE.

The interaction model (Conflict * Punishment Risk) showed an interaction between Conflict and Punishment Risk (χ^2^ (2) = 18.6, *p*<.001) (see Fig. 4). In line with prediction 3, follow up contrasts of the parameter estimates showed that the largest mean difference was between High Risk vs. No Risk during High Conflict (estimate: 0.13, *SE* = 0.07, *z* = 1.8, *p* = .035 [*one tailed*]). Furthermore, cEMG amplitude was lower for High Conflict than Low conflict for No Risk (estimate: 0.06, *SE* = 0.02, *z* = 2.33, *p = *.02). No other pair-wise contrasts were significant.

To summarize, our results provided support for predictions 1 and 2, and partial support for Prediction 3. Cognitive conflict strongly interacted with the time-course of the cEMG, where cEMG in the earlier time-bins was sensitive to Conflict and predictive of performance accuracy during High Conflict. In contrast to the High Risk, condition, the Low Risk condition had little impact on overall cEMG amplitude. In line with Prediction 3, the largest difference was between No vs. High Risk during High Conflict.

### Corrugator EMG: Prediction 4–7

To test Predictions 4 (error responses > correct responses), 5 (error responses during Punishment Risk > No Risk), 6 (error cEMG predicts post-error slowing), and 7 (error responses > correct responses driven by Punishment Risk) we fitted a series of models to the Post-response cEMG amplitude.

#### Prediction 4

**Figure 5:**
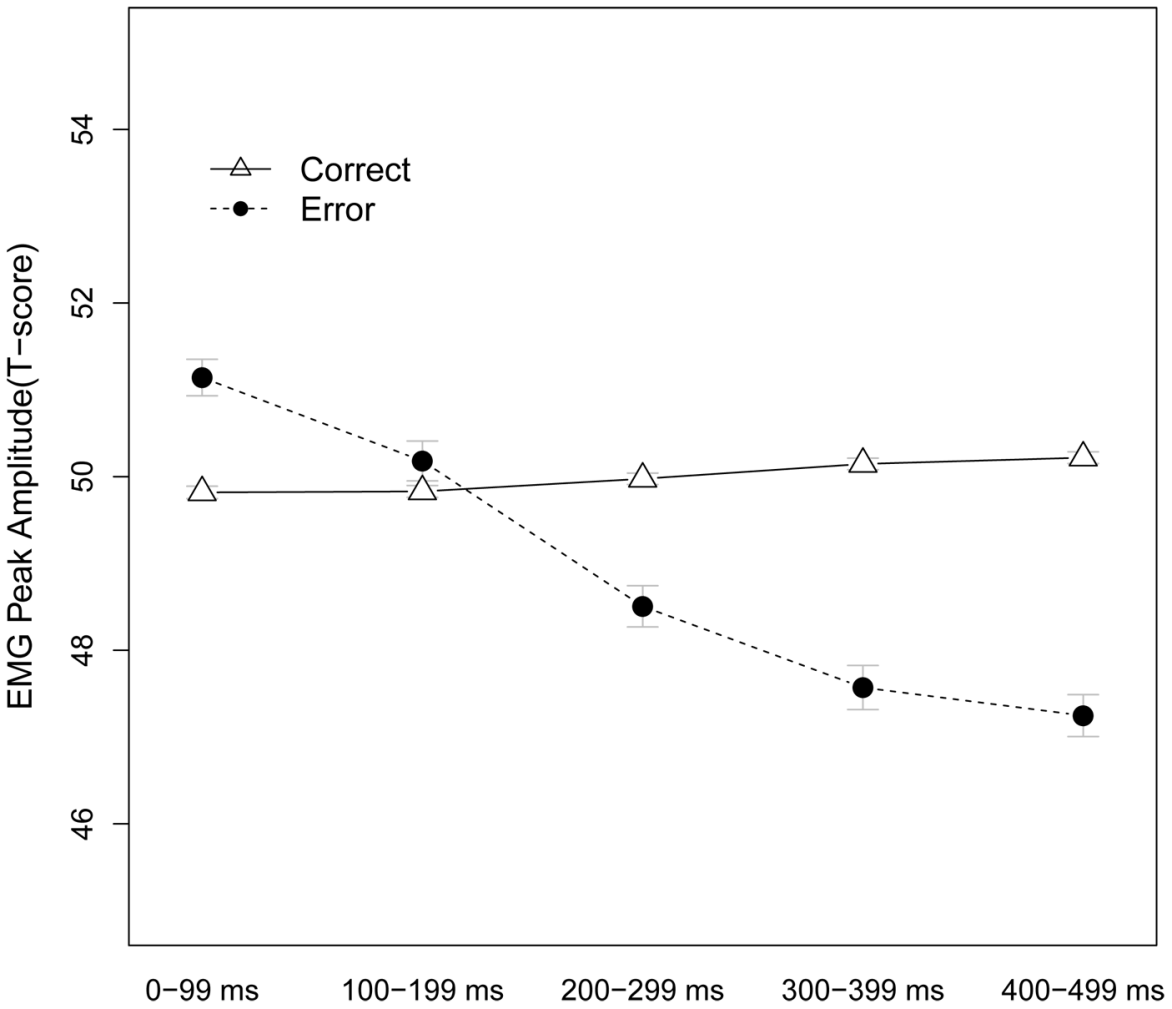
Time course of Error main effect Post-response. The cEMG was amplified directly within 0–99 ms following error responses (*p*<.05). Error bars denote SE.

The model, Accuracy (Correct/Error) * Time-bin (0–4), provided support for the prediction. The model showed a main effect of Accuracy (χ^2^ (1) = 4.27, *p* = .038), and a strong Accuracy x Time-bin interaction (χ^2^ (1) = 15.39, *p*<.001) (see Fig. 5). Simple effects showed that cEMG amplitude 0–99 ms following the response (i.e., at the intercept) was higher for Error responses relative to Correct responses (*β* = 0.19, *SE = *0.09, *z* = 2.1, p = .035). The slope of the Time-bin effect was negative for Error trials (*β* = −0.10, *SE* = 0.03, *z* = −3.93, *p*<.001), which was reversed for Correct trials (Time-bin x Accuracy: *β* = 0.11, *SE* = 0.03, *z* = 3.9, *p*<.001). As predicted (see Fig. 5), the cEMG was larger for Error than Correct responses within the first 100 ms, corresponding to the time course of the ERN [33].

To further examine this predicted effect, we limited the following analyses to the first time bin (0–99 ms). In order to rule out confounds unrelated to post-response cEMG, we used residualization to decorrelate the cEMG following the response (0–99 ms Post-response) from activity *prior* to the response (99-0 ms Pre-response). Consequently, the resulting residual cEMG Post-response vector is orthogonal to the Pre-response cEMG and thereby unaffected by any differences in Pre-response amplitude. A model with Accuracy as single predictor showed a main effect (χ^2^ (1) = 4.14, *p* = .042), where Error response (residual) cEMG amplitude was higher than Correct response (residual) cEMG amplitude (*β* = 0.14, *SE* = 0.067).

In sum, we found two kinds of support for Prediction 4; cEMG was amplified following response errors within 100 ms post-response, and the error-amplified cEMG was independent from the cEMG activity preceding the erroneous response.

#### Prediction 5

We fitted an Accuracy (Correct/Error) * Punishment Risk (No/Low/High) LMM to asses if post-response (residual) cEMG was modulated by the expected consequences of errors. The results based on non-residualized data were highly similar, but we focused here on the residualized EMG to fully control for any effects due to pre-response differences.

**Figure 6:**
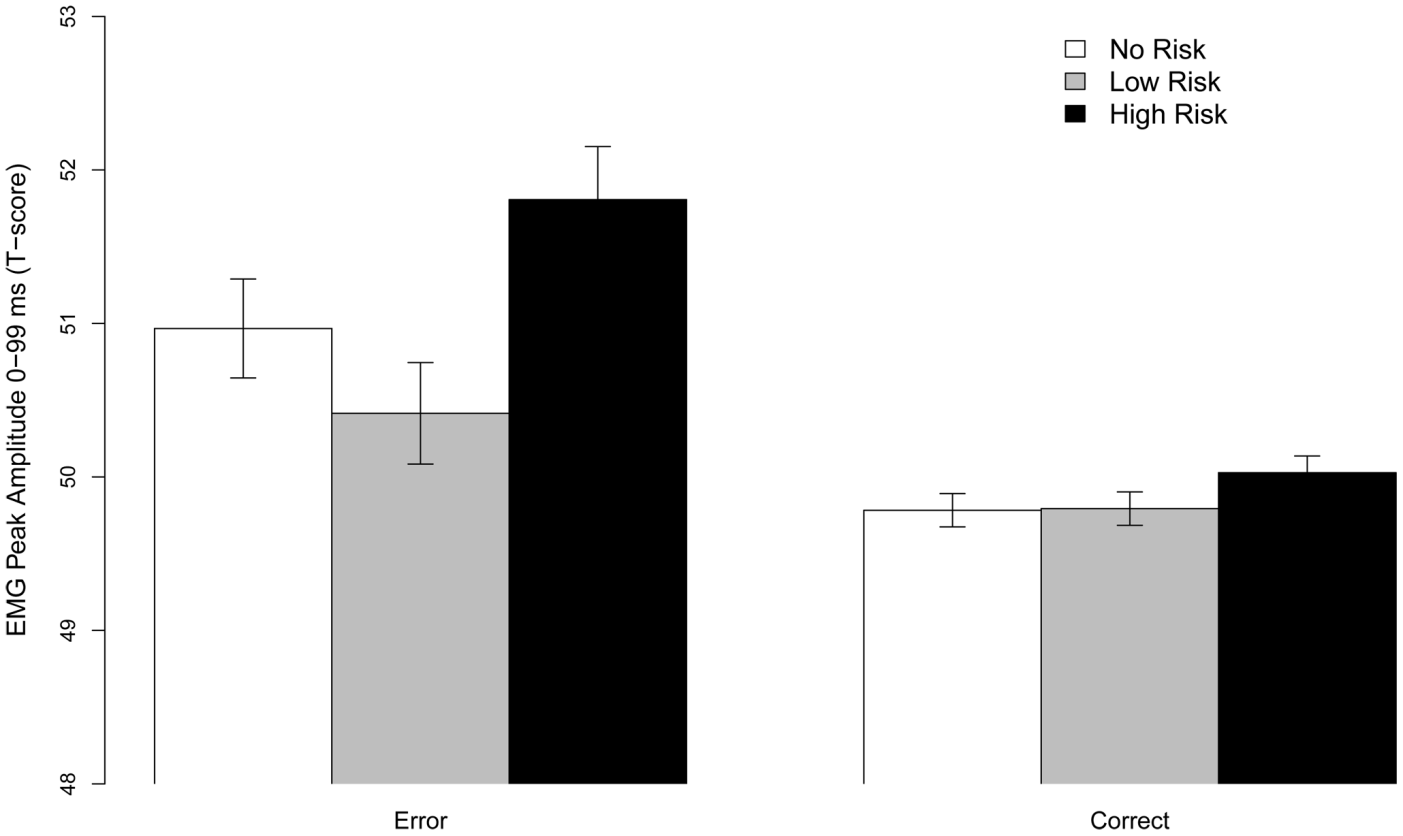
Error x Punishment Risk interaction within 0–99 ms after the behavioral response. The cEMG was amplified by High Risk following Errors but not Correct responses (*p*<.05). Error bars denote SE.

The model showed a main effect of Accuracy (χ^2^ (1) = 4.13, *p* = .042, a main effect of Punishment Risk (χ^2^ (2) = 7.92, *p* = .019), and critically, an Accuracy x Punishment Risk interaction (χ^2^ (2) = 8.57, *p* = .013) (See Fig. 6). Simple effects showed that this interaction was driven by a tendency to lower cEMG for Correct relative to Error responses for No Risk (*β* = −0.11, *SE* = 0.07, *z* = 1.57, *p* = .058 [*one tailed*]) and Low Risk (as indicated by the non-significant simple interaction; z = 0.63), which was amplified for High Risk (Accuracy x High PE: *β* = −0.10, *SE* = 0.05, *z* = −2.14, *p* = .03). Follow up contrasts of the parameter estimates showed that EMG amplitude following Errors was higher for High Risk than No Risk (estimate: 0.16, *SE* = 0.06, *z* = 2.64, *p* = .008) and Low Risk (estimate: 0.17, *SE* = 0.05, *z* = 3.55, *p*<.001). The Error EMG amplitude did not differ between No Risk and Low Risk (*z* = 0.12). Thus, in support of Prediction 5, error-amplified cEMG was enhanced by the perceived risk of punishment following errors.

#### Prediction 6: Post-error slowing

The ERN is often associated with subsequent performance adjustments, where a larger ERN on an error trial is related to slower performance on the following trial (Post-Error Slowing [33]). Therefore, we assessed if the Post-error cEMG was similarly functionally related to performance following errors. This prediction was supported: A model predicting RT (correct trials) from the accuracy and Post-response cEMG (0–99 ms residual) on the *preceding* trial (Pre-Accuracy: Error/Correct * Post-response cEMG) showed the predicted significant interaction (χ^2^ (1) = 7.39, *p* = .006). The interaction showed the predicted positive relation between slowing of RT and cEMG if the preceding trial was an Error (*β* = 6.02, *SE* = 2.28, *z = *2.64, *p* = .008), but not Correct (Post-response cEMG x Pre-Accuracy: *β* = −6.51, *SE* = 2.39, *z = *−2.72, *p* = .006). Thus, post-error cEMG is predictive of Post-Error slowing. The subsequent model including Punishment Risk showed no additional interactions (*p*s>.23). We also ran the corresponding analyses for post-error accuracy adjustments, and found no significant effects.

#### Prediction 7: Control group comparison

**Figure 7:**
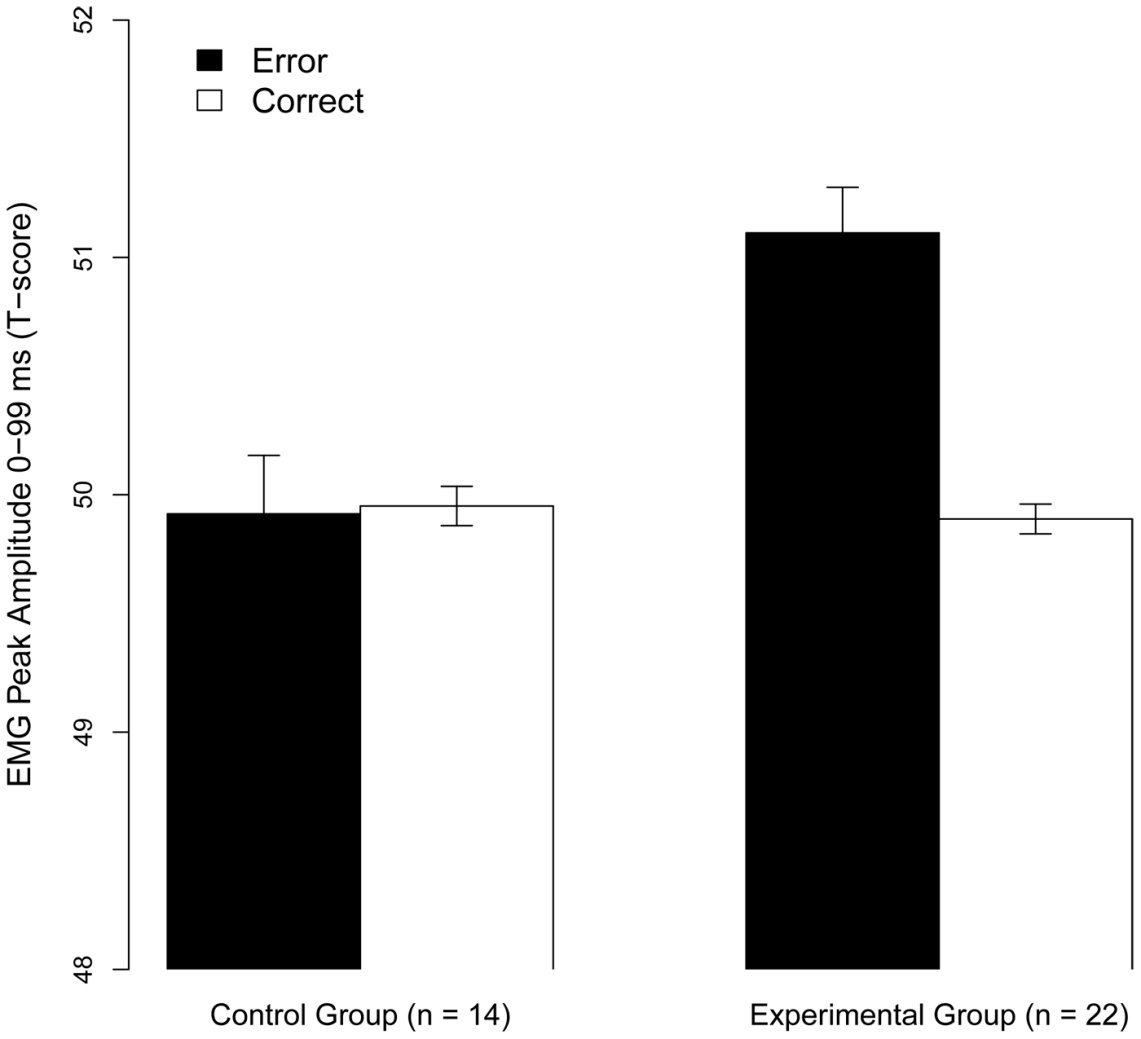
The cEMG was amplified following Errors in the Experimental, but not the Control, group (*p*<.0001). Error bars denote SE.

To validate that the modulation of Post-error cEMG by Punishment Risk was driven by the expected consequences of errors rather than shock anticipation or other factors unrelated to performance, we include a control group in the analysis (see Method for details). We analyzed post-response cEMG in the first time-bin (0–99 ms) with an Accuracy (Correct/Error) * Group (Control/Experimental) model. The model showed a main effect of Accuracy (χ^2^ (1) = 34.52, *p*<.001) and an Accuracy x Group interaction (χ^2^ (1) = 13.72, *p* = .0001). Simple effects showed that Accuracy had no significant effect for the Control group (*β* = 0.02, *SE = *0.02, *z* = 0.77, *p* = .22), that cEMG amplitude did not differ by Group for Correct responses (*β* = −0.01, *SE* = 0.01, *z* = −0.65, *p* = .515), while, critically, the Experimental group x Error interaction (*β* = 0.12, *SE* = 0.03, *z* = 3.70, *p*<.001) showed that the effect of Errors was amplified for the Experimental group (see Fig. 7). Thus, as predicted, the amplification of cEMG by response errors was directly related to the expected consequences of error.

## Discussion

The present study had two main objectives; to characterize how avoidance motivation impacts cognitive control through a parametric manipulation of perceived *punishment risk*, and (2) investigate the cEMG as a novel index of this process by means of testing a set of specific hypotheses derived from recent work on the neural underpinnings of motivated cognitive control [21]. First, as predicted, we showed that the perceived risk of punishment for error commissions attenuated the detrimental effect of high response conflict on performance. This effect was non-linear: Low Risk enhanced performance, while High Risk had little effect. Second, the cEMG was highly sensitive to both punishment risk and response conflict, showing the predicted properties of a signal that integrates cognitive control demands and avoidance motivation. In particular, the present study provides the first report of cEMG activity as a correlate of error monitoring, and shows that this correlate (i) operates on a similar time-scale as the ERP index of error processing, the ERN, (ii) is modulated by the expected aversive consequences of errors, and (iii) related to post-error slowing.

### Behavioral results

The effect of punishment risk on behavior was dependent on the level of response conflict. Whereas Low Risk impaired performance in the Low Conflict condition, the reverse was true for High Conflict (see Fig. 2). Together with the pattern of RT results (see Fig. 3), which showed response slowing at Low Risk during the Low Conflict condition and faster responses in the High Conflict condition for both Low and High Punishment Risk, the accuracy results indicate that participants traded Low Conflict accuracy for higher accuracy during the difficult High Conflict condition. Viewed in a framework where evidence for each choice is gradually and stochastically accumulated over time, such a trade-off could be implemented pro-actively either by changing the starting point for evidence accumulation (i.e., akin to a shift in the subjective probabilities of each target type) or lowering the decision boundary for High Conflict targets, both which would predict more errors during Low Conflict [53]. This trade-off between Low and High Conflict accuracy during Low Risk blocks indicate that punishment risk affected proactive, rather than reactive cognitive control [54]. Proactive control, where a strategy is implemented prior to control demands, can be contrasted against reactive, “just-in-time” control (e.g., the last moment stop response to a red traffic light) [40], [55], where the former is considered more relevant for goal-directed behavior [55]. Reactive control, which would only be initiated at the onset of High Conflict targets, should not affect the processing of Low Conflict targets, which none the less occurred in the present study. This conclusion is strengthened by the fact that punishment risk did not affect post-error slowing and intra-trial adjustments (as indexed by cEMG, see Results), both commonly thought to index reactive cognitive control that is elicited by performance errors or detection of conflict [56]. Similar proactive cautionary behavior in a Go/No-Go task has been described in a task where errors were punished by monetary loss, and was argued to be reflected by sustained, rather than phasic, ACC activity [18], suggesting that the ACC/aMCC proactively modulates behavior to avoid punishment [21]. More generally, the suggestion that cognitive control is a system that enables organisms to proactively alter behavior to avoid aversive outcomes [35], [57] and thereby reduce uncertainty in the service of optimal behavior [58] underscores the significance of our behavioral findings.

The effect of punishment risk on performance accuracy could be formally described by an inverted-U shape during High Conflict, as performance was facilitated by Low Risk, while High Risk had no reliable effect on performance (see Fig. 2). This effect resembles the classical Yerkes-Dodson law, which states that arousal impact performance efficiency according to an inverted-U shape [59]. This relation between external demands and performance is often termed “choking under the pressure” [60]. For example, an athlete can be more prone to miss a well-trained throw in a high-stake match rather than in a practice match at the home arena. Commonly, choking under pressure is explained either by resource consumption (e.g., of working memory) or distraction [60]. Distraction effects are most likely to be produced under outcome-pressure, for instance if the consequences of errors are aversive [60]. A possible neurophysiological account of such distraction is provided by Aston-Jones and Cohen [61], who posits that the locus coeruleus – norephinephrine (LC-NE) system regulates arousal to optimize performance. Tonic LC-NE activity has an inverted-U like effect of performance, where elevated tonic NE levels are related to increased distractibility and errors commissions [60]. The ACC regulate the LC-NE based on the utility of current behavior, serving to optimize performance following, for example, error commissions. The theory predicts that prolonged disutility, which could supposedly be exemplified by repeated errors commissions when errors are costly, will drive the LC-NE system into the tonic mode [60]. One might speculate that High Risk might have driven the LC-NE system into an elevated tonic state, possibly because repeated errors despite high risk of punishment in a given context might serve as an imperative signal to exchange that context for a more beneficial one. Our SCL findings, which showed that punishment risk had a strong and linear effect on physiological arousal, are in line with this proposal (see also [62]). An important goal with future studies should be to fully describe the relationship between cognitive control and punishment risk by using a more fine-grained parametric manipulation of punishment risk.

### Corrugator EMG Results

We hypothesized that activity in the corrugator superscilii muscle would index the integration of cognitive control and avoidance motivation. This hypothesis was based on (1) similarities between the previously described response properties of the cEMG and neural activity in the aMCC [21], and (2) the influence of the aMCC on the corrugator supercilii muscle via projections from the facial nucleus [21], [25]. Our hypothesis was specified by the formulation of 7 detailed predictions that were tested. All seven predictions received support, and are discussed sequentially below.

As stated in Prediction 1, cEMG was amplified for High Conflict, but this effect was most evident in the early time-course in trials with longer RT. Furthermore, the amplified cEMG predicted performance accuracy during High, but not Low Conflict trials. Thus, the pre-response cEMG likely reflected within-trial performance adjustments when response conflict was high. One of the most prominent theories of ACC function, the Conflict Monitoring theory, argues that pre-response ACC activity in conflict conditions (the N2 ERP) is directly related to post-response activity following errors (the ERN), suggesting that both reflect the activity of a system monitoring conflict between response tendencies [39]. Both components are responsive to conflict and share a common neural-generator in the ACC. The N2 potential is seen prior to responses on correct trials with high conflict, and thus reflects the timely resolution of conflict. In contrast, the ERN which follows an erroneous response reflects the very conflict that led to an error being elicited. The time to respond in correct trials (i.e., RT) has been shown to be directly proportional to the amount of conflict, as the response is delayed by the higher levels of conflict [39]. This fits well with the temporal characteristics of the pre-response cEMG on correct trials in our results, where the effect of conflict was visible only during high conflict trials with longer RT. Further support for this interpretation was that the amplified pre-response cEMG during High Conflict directly predicted behavioral accuracy [39].

In accordance with our Prediction 2, average cEMG was sensitive to punishment risk. However, rather than showing a linear increase with punishment risk level (No < Low < High), cEMG was amplified only for High Risk (No/Low < High). The amplification of cEMG by punishment risk is in concordance with previous findings using a threat-of-shock paradigm [20], [63] and the substantial body of research relating cEMG activity to negative emotions [64]. Interestingly, the non-linear effect of punishment risk on cEMG dissociates it from SCL which increased linearly with punishment risk level. However, the cause of this dissociation is currently unknown.

As stated in Prediction 3, punishment risk and response conflict interacted in the effect on cEMG, with the largest difference between High Risk and No Risk during High Conflict (see Fig. 4). However, these results were complicated by the fact that activity was lower for High than Low Conflict during No Risk, whereas there was no difference between Low and High Conflict during Punishment Risk blocks. Previously, high conflict No-Go trials have been shown to elicit amplified cEMG activity compared to low conflict Go trials [32], but not in the Simon conflict task [65]. Taken together with the present findings, this suggests that additional factors, such as the within-trial time course (see above) or other task-specific parameters, might modulate the effect of conflict on cEMG activity.

In sum, our results largely support Prediction 1–3; cEMG was responsive to High Conflict (Prediction 1), especially early in the within-trial time-course. Importantly, this pattern of results can be accounted for by established theory. Furthermore, cEMG was responsive to punishment risk (Prediction 2), and showed the hypothesized difference between No and High Risk during High Conflict (Prediction 3).

We derived predictions 4–7 from the properties of a known index of motivated cognitive control; the ERN [38]. Specifically, we hypothesized that cEMG should be higher following response errors than correct responses (Prediction 4); that this effect should be potentiated by Punishment Risk (Prediction 5); predictive of Post-error slowing (Prediction 6); and directly related to the expected consequences of errors (Prediction 7). In line with prediction 4, cEMG was reliably higher for errors than correct responses within the first 100 ms following the behavioral response (see Fig. 5). This difference was robust also when statistically controlling for the cEMG level prior to the response. The ERN also peaks between 50 and 100 ms following errors [33], which indicate that the error-potentiated cEMG is unlikely to reflect processes “down-stream” of the ERN, such as a secondary conscious reaction to errors. Rather, given that the corrugator supercilii muscle in part is innervated by the ACC/aMCC via the brainstem facial nucleus [26], the temporal concurrence of the ERN and the error cEMG suggest that they might share a common generator, possibly in the aMCC [21]. Obviously, the lack of simultaneous EMG and EEG recordings in the present study precluded us from conclusively establishing this link. The similarity between the error cEMG and the ERN was further reinforced by the shared relation to post-error slowing (prediction 6). Gehring and colleagues [33] showed that the magnitude of the ERN predicted the amount of behavioral slowing on the following trial, which was interpreted to reflect the strategic recruitment of control (but see [66]) for a contrasting view on the mechanisms underlying post-error slowing). Similarly, the cEMG activity following errors, but not correct responses, significantly predicted longer RT on the following correct trial. The cEMG activity did not predict post-error accuracy, in line with recent literature arguing that post-error slowing and post-error accuracy increases can occur independently [67]

The ERN is modulated by the motivational value of error commissions [38]. For example, errors punished with loss of money [68] or an aversive noise [37] elicits a stronger ERN [39]. Such findings provide critical support for the TACH [21], as they indicate that information about cognitive conflict or response errors is integrated in the ACC/aMCC with information related to the motivational value of different actions. Based on these premises, we expected (Prediction 5) that punishment risk would potentiate the error-amplified cEMG. In support of this prediction, error cEMG was most pronounced during High Risk blocks, and differentiated from both No Risk and Low Risk (See. Fig. 6). In fact, pair-wise contrasts indicated that cEMG only was reliably larger for erroneous as compared to correct responses during high punishment risk The lack of a general main effect of error across levels of punishment risk might reflect either a threshold effect (i.e., that cEMG is a relatively insensitive proxy for brain-based error-monitoring processes and only High Risk elicited strong enough post-error activity to be visible in the cEMG), or that the cEMG primarily indexes the integration of negative affect and error monitoring rather than all aspects of cognitive control. Our results support the former explanation as there was no effect of punishment risk on pre-response cEMG. An alternative possibility is that the participants in the control group learned that errors had absolutely no consequences, as they were explicitly informed that there was no contingency between their performance and the shocks they would receive. This might differ from how errors are interpreted in real-world settings, where errors typically have some form of consequences, either internal or external [69]. This possibility could be addressed by a replication experiment without an external manipulation of motivation. Such an experiment would further clarify the relation between the ERN and the error-potentiated cEMG.

Finally, we included a critical control group to confirm that the error cEMG was driven by the expected aversive consequences of errors, rather than shock anticipation or general anxiety (Prediction 7). Both groups performed the identical experimental task as in the main experiment, with one critical difference; the control group knew that errors had no bearing on the amount of shocks they received. As predicted, only the experimental group showed error-potentiated cEMG (see Fig. 7), indicating that the effect was driven by the expected aversive consequences of error commissions, rather than anticipation of pain or anxiety per se. Funneled interviews indicated that all participants in the experimental group believed there was a contingency between their performance and the amount of punishment they received, whereas the Control group reported no contingency between their performance and the amount of punishment (see Method for details). Based on the results of our control experiment and the post-experimental interviews, we conclude that the group-level effects of punishment risk on post-error cEMG likely reflects the proactive motivation to avoid aversive consequences, rather than being driven by mere pain anxiety, which should have been equal for both groups.

A possible caveat concerns how specific these effects are for the corrugator supercilii, or if also other facial muscles (e.g., the zygomaticus major) would exhibit a similar pattern of activation. We consider this to be unlikely for several reasons. First, a large literature show that the corrugater supercilii, but not the zygomaticus major or other muscles in the lower part of the face, is activated by aversive images [64] and threat of shock [20], [63]. Second, the muscles of the lower face are innervated by different brain regions than the muscles of the upper face (including the corrugator supercilii) [26], which together with our anatomically grounded apriori hypothesis (see “The Present Study”) about the functional overlap between the cEMG response and the ERN, suggest considerable specificity in the response pattern of the corrugator muscle to punishment risk and cognitive control. Even so, an important goal for future studies is to investigate convergence and divergence between different measures related to punishment risk and cognitive control.

In summary, the post-response cEMG conformed to the predicted properties of a signal that integrates cognitive control and avoidance motivation in striking resembles to the ERN (prediction 4), both regarding time-course, relation to post-error slowing, and sensitivity to error consequences (prediction 5–7). In concert with the support for predictions 1–3 (see above), these results strongly suggest that cEMG might reflect the integration of cognitive control and avoidance motivation, and thus possibly aMCC activity, as described by TACH. Further confirmation of this hypothesis is however needed. In particular, concurrent cEMG and EEG/fMRI recordings are critical to conclusively tie cEMG and aMCC activity during potentially dangerous situations when control is at demand. However, these preliminary results are in line with TACH [21], as they provide new evidence for functional convergence of cognitive conflict and avoidance motivation, and that the corrugator supercilii, innervated by the aMCC, is sensitive to this convergence.

To conclude, the current study showed that aversive motivation has a substantial impact on cognitive control behavior. This finding stresses the importance of considering not only the goals of behavior, but also the potentially aversive or harmful consequences of failure, to fully characterize cognitive control. We also for the first time demonstrate that cEMG is sensitive to the interaction of cognitive control and avoidance motivation. This finding is concordant with the proposed integrative role of the aMCC in potentially dangerous and control demanding situations [21], and suggest that further investigation of the response properties of the cEMG might be highly fruitful.

## References

[1] Mansouri FA, Tanaka K, Buckley MJ (2009) Conflict-induced behavioural adjustment: a clue to the executive functions of the prefrontal cortex. Nature reviews Neuroscience 10: 141–152 doi:10.1038/nrn2538 19153577 10.1038/nrn2538

[2] Miller EK, Cohen JD (2001) An integrative theory of prefrontal cortex function. Annual review of neuroscience 24: 167–202 doi:10.1146/annurev.neuro.24.1.167 10.1146/annurev.neuro.24.1.167 11283309

[3] MacDonald KB (2008) Effortful control, explicit processing, and the regulation of human evolved predispositions. Psychological review 115: 1012–1031 doi:10.1037/a0013327 18954212 10.1037/a0013327

[4] Davidson RJ (2002) Anxiety and affective style: role of prefrontal cortex and amygdala. Biological Psychiatry 51: 68–80 doi:10.1016/S0006-3223(01)01328-2 11801232 10.1016/s0006-3223(01)01328-2

[5] Heatherton TF, Wagner DD (2011) Cognitive neuroscience of self-regulation failure. Trends in cognitive sciences 15: 132–139 doi:10.1016/j.tics.2010.12.005 21273114 10.1016/j.tics.2010.12.005 PMC3062191

[6] Braver TS, Paxton JL, Locke HS, Barch DM (2009) Flexible neural mechanisms of cognitive control within human prefrontal cortex. Proceedings of the National Academy of Sciences 106: 7351–7356 doi:10.1073/pnas.0808187106 10.1073/pnas.0808187106 PMC2678630 19380750

[7] Jimura K, Locke HS, Braver TS (2010) Prefrontal cortex mediation of cognitive enhancement in rewarding motivational contexts. Proceedings of the National Academy of Sciences of the United States of America 107: 8871–8876 doi:10.1073/pnas.1002007107 20421489 10.1073/pnas.1002007107 PMC2889311

[8] Kouneiher F, Charron S, Koechlin E (2009) Motivation and cognitive control in the human prefrontal cortex. Nature neuroscience 12: 939–945 doi:10.1038/nn.2321 19503087 10.1038/nn.2321

[9] Pessoa L (2009) How do emotion and motivation direct executive control? Trends in cognitive sciences 13: 160–166 doi:10.1016/j.tics.2009.01.006 19285913 10.1016/j.tics.2009.01.006 PMC2773442

[10] Locke HS, Braver TS (2008) Motivational influences on cognitive control: Behavior, brain activation, and individual differences. Cognitive, Affective, & Behavioral Neuroscience 8: 99–112 doi:10.3758/CABN.8.1.99 10.3758/cabn.8.1.99 18405050

[11] Schouppe N, De Houwer J, Ridderinkhof KR, Notebaert W (2012) Conflict: run! Reduced Stroop interference with avoidance responses. Quarterly journal of experimental psychology (2006) 65: 1052–1058 doi:10.1080/17470218.2012.685080 22640724 10.1080/17470218.2012.685080

[12] Lim S-L, Padmala S, Pessoa L (2009) Segregating the significant from the mundane on a moment-to-moment basis via direct and indirect amygdala contributions. Proceedings of the National Academy of Sciences of the United States of America 106: 16841–16846 doi:10.1073/pnas.0904551106 19805383 10.1073/pnas.0904551106 PMC2757860

[13] Glimcher PW (2011) Understanding dopamine and reinforcement learning: the dopamine reward prediction error hypothesis. Proceedings of the National Academy of Sciences of the United States of America 108 (Suppl) 15647–15654 doi:10.1073/pnas.1014269108 21389268 10.1073/pnas.1014269108 PMC3176615

[14] Phelps EA (2006) Emotion and cognition: insights from studies of the human amygdala. Annual review of psychology 57: 27–53 doi:10.1146/annurev.psych.56.091103.070234 10.1146/annurev.psych.56.091103.070234 16318588

[15] Dayan P, Huys QJM (2009) Serotonin in affective control. Annual review of neuroscience 32: 95–126 doi:10.1146/annurev.neuro.051508.135607 10.1146/annurev.neuro.051508.135607 19400722

[16] Boureau Y-L, Dayan P (2011) Opponency revisited: competition and cooperation between dopamine and serotonin. Neuropsychopharmacology: official publication of the American College of Neuropsychopharmacology 36: 74–97 doi:10.1038/npp.2010.151 20881948 10.1038/npp.2010.151 PMC3055522

[17] Dayan P, Seymour B (2008) Values and Actions in Aversion. In: Glimcher PW, Camerer C, Fehr E, Poldrack RA, editors. Neuroeconomics: Decision making and the brain. Academic Press. pp. 175–192.

[18] Simões-Franklin C, Hester R, Shpaner M, Foxe JJ, Garavan H (2010) Executive function and error detection: The effect of motivation on cingulate and ventral striatum activity. Human brain mapping 31: 458–469 doi:10.1002/hbm.20879 19718655 10.1002/hbm.20879 PMC4485396

[19] Robinson OJ, Letkiewicz AM, Overstreet C, Ernst M, Grillon C (2011) The effect of induced anxiety on cognition: threat of shock enhances aversive processing in healthy individuals. Cognitive, affective & behavioral neuroscience 11: 217–227 doi:10.3758/s13415-011-0030-5 10.3758/s13415-011-0030-5 PMC3169349 21484411

[20] Shackman AJ, Maxwell JS, McMenamin BW, Greischar LL, Davidson RJ (2011) Stress potentiates early and attenuates late stages of visual processing. The Journal of neuroscience: the official journal of the Society for Neuroscience 31: 1156–1161 doi:10.1523/JNEUROSCI.3384-10.2011 21248140 10.1523/JNEUROSCI.3384-10.2011 PMC3037336

[21] Shackman AJ, Salomons TV, Slagter Ha, Fox AS, Winter JJ, et al (2011) The integration of negative affect, pain and cognitive control in the cingulate cortex. Nature reviews Neuroscience 12: 154–167 doi:10.1038/nrn2994 21331082 10.1038/nrn2994 PMC3044650

[22] Ridderinkhof KR, Ullsperger M, Crone EA, Nieuwenhuis S (2004) The role of the medial frontal cortex in cognitive control. Science (New York, NY) 306: 443–447 doi:10.1126/science.1100301 10.1126/science.1100301 15486290

[23] Etkin A, Egner T, Kalisch R (2011) Emotional processing in anterior cingulate and medial prefrontal cortex. Trends in cognitive sciences 15: 85–93 doi:10.1016/j.tics.2010.11.004 21167765 10.1016/j.tics.2010.11.004 PMC3035157

[24] Duerden EG, Albanese M-C (2011) Localization of pain-related brain activation: A meta-analysis of neuroimaging data. Human brain mapping doi:10.1002/hbm.21416 10.1002/hbm.21416 PMC6869965 22131304

[25] Morecraft RJ, Louie JL, Herrick JL, Stilwell-Morecraft KS (2001) Cortical innervation of the facial nucleus in the non-human primate: a new interpretation of the effects of stroke and related subtotal brain trauma on the muscles of facial expression. Brain: a journal of neurology 124: 176–208. 11133797 10.1093/brain/124.1.176

[26] Morecraft RJ, Stilwell-Morecraft KS, Rossing WR (2004) The motor cortex and facial expression: new insights from neuroscience. The neurologist 10: 235–249. 15335441 10.1097/01.nrl.0000138734.45742.8d

[27] Burrows AM (2008) The facial expression musculature in primates and its evolutionary significance. BioEssays: news and reviews in molecular, cellular and developmental biology 30: 212–225 doi:10.1002/bies.20719 10.1002/bies.20719 18293360

[28] Burrows AM, Waller BM, Parr LA (2009) Facial musculature in the rhesus macaque (Macaca mulatta): evolutionary and functional contexts with comparisons to chimpanzees and humans. Journal of anatomy 215: 320–334 doi:10.1111/j.1469-7580.2009.01113.x 19563473 10.1111/j.1469-7580.2009.01113.x PMC2750044

[29] Larsen JT, Norris CJ, Cacioppo JT (2003) Effects of positive and negative affect on electromyographic activity over zygomaticus major and corrugator supercilii. Psychophysiology 40: 776–785. 14696731 10.1111/1469-8986.00078

[30] Dimberg U, Thunberg M, Elmehed K (2000) Unconscious facial reactions to emotional facial expressions. Psychological science 11: 86–89. 11228851 10.1111/1467-9280.00221

[31] Prkachin KM (1992) The consistency of facial expressions of pain: a comparison across modalities. Pain 51: 297–306. 1491857 10.1016/0304-3959(92)90213-U

[32] Schacht A, Nigbur R, Sommer W (2009) Emotions in Go/NoGo conflicts. Psychological research 73: 843–856 doi:10.1007/s00426-008-0192-0 19030874 10.1007/s00426-008-0192-0

[33] Gehring WJ, Goss B, Coles MGH, Meyer DE, Donchin E (1993) A neural system for error detection and compensation. Psychological Science 4: 385–390 doi:10.1111/j.1467-9280.1993.tb00586.x

[34] Holroyd CB, Coles MGH (2002) The neural basis of human error processing: reinforcement learning, dopamine, and the error-related negativity. Psychological review 109: 679–709. 12374324 10.1037/0033-295X.109.4.679

[35] Botvinick MM, Braver TS, Barch DM, Carter CS, Cohen JD (2001) Conflict Monitoring and Cognitive Control 108: 624–652 doi:10.1037//0033-295X.I08.3.624 10.1037/0033-295x.108.3.624 11488380

[36] Hajcak G, Moser JS, Yeung N, Simons RF (2005) On the ERN and the significance of errors. Psychophysiology 42: 151–160 doi:10.1111/j.1469-8986.2005.00270.x 15787852 10.1111/j.1469-8986.2005.00270.x

[37] Riesel A, Weinberg A, Endrass T, Kathmann N, Hajcak G (2012) Punishment has a lasting impact on error-related brain activity. Psychophysiology 49: 239–247 doi:10.1111/j.1469-8986.2011.01298.x 22092041 10.1111/j.1469-8986.2011.01298.x

[38] Hajcak G (2012) What We've Learned From Mistakes: Insights From Error-Related Brain Activity. Current Directions in Psychological Science 21: 101–106 doi:10.1177/0963721412436809

[39] Yeung N, Botvinick MM, Cohen JD (2004) The neural basis of error detection: conflict monitoring and the error-related negativity. Psychological review 111: 931–959 doi:10.1037/0033-295X.111.4.939 15482068 10.1037/0033-295x.111.4.939

[40] Braver TS (2012) The variable nature of cognitive control: a dual mechanisms framework. Trends in cognitive sciences 16: 106–113 doi:10.1016/j.tics.2011.12.010 22245618 10.1016/j.tics.2011.12.010 PMC3289517

[41] Brown JW, Braver TS (2007) Risk prediction and aversion by anterior cingulate cortex. Cognitive, affective & behavioral neuroscience 7: 266–277. 10.3758/cabn.7.4.266 18189000

[42] Calvo MG, Lundqvist D (2008) Facial expressions of emotion (KDEF): identification under different display-duration conditions. Behavior research methods 40: 109–115. 18411533 10.3758/brm.40.1.109

[43] Fridlund AJ, Cacioppo JT (1986) Guidelines for human electromyographic research. Psychophysiology 23: 567–589. 3809364 10.1111/j.1469-8986.1986.tb00676.x

[44] Simmonds DJ, Pekar JJ, Mostofsky SH (2008) Meta-analysis of Go/No-go tasks demonstrating that fMRI activation associated with response inhibition is task-dependent. Neuropsychologia 46: 224–232 doi:10.1016/j.neuropsychologia.2007.07.015 17850833 10.1016/j.neuropsychologia.2007.07.015 PMC2327217

[45] Kenner NM, Mumford JA, Hommer RE, Skup M, Leibenluft E, et al (2010) Inhibitory motor control in response stopping and response switching. The Journal of neuroscience: the official journal of the Society for Neuroscience 30: 8512–8518 doi:10.1523/JNEUROSCI.1096-10.2010 20573898 10.1523/JNEUROSCI.1096-10.2010 PMC2905623

[46] Mostofsky SH, Simmonds DJ (2008) Response inhibition and response selection: two sides of the same coin. Journal of cognitive neuroscience 20: 751–761 doi:10.1162/jocn.2008.20500 18201122 10.1162/jocn.2008.20500

[47] Gomez P, Ratcliff R, Perea M (2007) A model of the go/no-go task. Journal of experimental psychology General 136: 389–413 doi:10.1037/0096-3445.136.3.389 17696690 10.1037/0096-3445.136.3.389 PMC2701630

[48] Bates D, Sarkar D (2007) lme4: Linear mixed-effects models using S4 classes.

[49] Jaeger TF (2008) Categorical Data Analysis: Away from ANOVAs (transformation or not) and towards Logit Mixed Models. Journal of memory and language 59: 434–446 doi:10.1016/j.jml.2007.11.007 Last Accessed 2013-05-31 19884961 10.1016/j.jml.2007.11.007 PMC2613284

[50] Baayen RH, Davidson DJ, Bates DM (2008) Mixed-effects modeling with crossed random effects for subjects and items. Journal of Memory and Language 59: 390–412 doi:10.1016/j.jml.2007.12.005

[51] Fox, John & Weisberg S (2011) An R Companion to Applied Regression. Thousand Oaks, CA: Sage.

[52] Bolker BM, Brooks ME, Clark CJ, Geange SW, Poulsen JR, et al (2009) Generalized linear mixed models: a practical guide for ecology and evolution. Trends in ecology & evolution 24: 127–135 doi:10.1016/j.tree.2008.10.008 19185386 10.1016/j.tree.2008.10.008

[53] Ratcliff R, McKoon G (2008) The diffusion decision model: theory and data for two-choice decision tasks. Neural computation 20: 873–922 doi:10.1162/neco.2008.12-06-420 18085991 10.1162/neco.2008.12-06-420 PMC2474742

[54] Chiew KS, Braver TS (2011) Positive affect versus reward: emotional and motivational influences on cognitive control. Frontiers in psychology 2: 279 doi:10.3389/fpsyg.2011.00279 22022318 10.3389/fpsyg.2011.00279 PMC3196882

[55] Aron AR (2011) From reactive to proactive and selective control: developing a richer model for stopping inappropriate responses. Biological psychiatry 69: e55–68 doi:10.1016/j.biopsych.2010.07.024 20932513 10.1016/j.biopsych.2010.07.024 PMC3039712

[56] Braver TS (2012) The variable nature of cognitive control: a dual mechanisms framework. Trends in cognitive sciences 16: 106–113 doi:10.1016/j.tics.2011.12.010 22245618 10.1016/j.tics.2011.12.010 PMC3289517

[57] Dreisbach G, Fischer R (2012) Conflicts as aversive signals. Brain and cognition 78: 94–98 doi:10.1016/j.bandc.2011.12.003 22218295 10.1016/j.bandc.2011.12.003

[58] Mushtaq F, Bland AR, Schaefer A (2011) Uncertainty and cognitive control. Frontiers in psychology 2: 249 doi:10.3389/fpsyg.2011.00249 22007181 10.3389/fpsyg.2011.00249 PMC3184613

[59] Teigen KH (1994) Yerkes-Dodson: A Law for all Seasons. Theory & Psychology 4: 525–547 doi:10.1177/0959354394044004

[60] DeCaro MS, Thomas RD, Albert NB, Beilock SL (2011) Choking under pressure: multiple routes to skill failure. Journal of experimental psychology General 140: 390–406 doi:10.1037/a0023466 21574739 10.1037/a0023466

[61] Aston-Jones G, Cohen JD (2005) An integrative theory of locus coeruleus-norepinephrine function: adaptive gain and optimal performance. Annual review of neuroscience 28: 403–450 doi:10.1146/annurev.neuro.28.061604.135709 10.1146/annurev.neuro.28.061604.135709 16022602

[62] Carp J, Compton RJ (2009) Alpha power is influenced by performance errors. Psychophysiology 46: 336–343 doi:10.1111/j.1469-8986.2008.00773.x 19207203 10.1111/j.1469-8986.2008.00773.x

[63] Bradley MM, Moulder B, Lang PJ (2005) When good things go bad: the reflex physiology of defense. Psychological science 16: 468–473 doi:10.1111/j.0956-7976.2005.01558.x 15943673 10.1111/j.0956-7976.2005.01558.x

[64] Lang PJ, Greenwald MK, Bradley MM, Hamm AO (1993) Looking at pictures: affective, facial, visceral, and behavioral reactions. Psychophysiology 30: 261–273. 8497555 10.1111/j.1469-8986.1993.tb03352.x

[65] Schacht A, Dimigen O, Sommer W (2010) Emotions in cognitive conflicts are not aversive but are task specific. Cognitive, affective & behavioral neuroscience 10: 349–356 doi:10.3758/CABN.10.3.349 10.3758/CABN.10.3.349 20805536

[66] Notebaert W, Houtman F, Opstal FV, Gevers W, Fias W, et al (2009) Post-error slowing: an orienting account. Cognition 111: 275–279 doi:10.1016/j.cognition.2009.02.002 19285310 10.1016/j.cognition.2009.02.002

[67] Danielmeier C, Ullsperger M (2011) Post-error adjustments. Frontiers in psychology 2: 233 doi:10.3389/fpsyg.2011.00233 21954390 10.3389/fpsyg.2011.00233 PMC3173829

[68] Hajcak G, Nieuwenhuis S, Ridderinkhof KR, Simons RF (2005) Error-preceding brain activity: robustness, temporal dynamics, and boundary conditions. Biological psychology 70: 67–78 doi:10.1016/j.biopsycho.2004.12.001 16168251 10.1016/j.biopsycho.2004.12.001

[69] Aarts K, De Houwer J, Pourtois G (2012) Evidence for the automatic evaluation of self-generated actions. Cognition 124: 117–127 doi:10.1016/j.cognition.2012.05.009 22687531 10.1016/j.cognition.2012.05.009

